# Investigating the Effects of Oral Ginseng on the Cancer-Related Fatigue and Quality of Life in Patients with Non-Metastatic Cancer

**Published:** 2018-10-01

**Authors:** Khatereh Pourmohamadi, Ahmad Ahmadzadeh, Mahmood Latifi

**Affiliations:** Health Research Institute, Thalassemia and Hemoglobinopathies Research Center, Ahvaz Jundishapur University of Medical Sciences, Ahvaz, Iran

**Keywords:** Ginseng, Cancer-related fatigue, Beck test

## Abstract

**Background:** Cancer affects the physical, psychological, and social aspects of the patients’ life. Cancer-related fatigue (CRF) is the most common and severe condition among cancer patients. Ginseng has long been used as an efficient treatment for CRF and improvement of quality of life (QOL). The present study aims to assess the efficacy of Panax Ginseng (PG) in reducing CRF in patients with non-metastatic cancer. In addition, the safety of the medication is evaluated.

**Materials and Methods:** This was a prospective clinical trial conducted on the patients (n=113) suffering from non-metastatic colon cancer (age range: 20-70 years old) referring to the Shafa Hospital, Ahvaz, Iran for chemotherapy treatment. After the chemotherapy sessions, the patients were randomly divided into two groups. The first group received daily dose of 100 mg PG for 30 days and the second group received placebo medication. The demographic information and clinical parameters of the patients including age, sex, weight, symptoms of fatigue, depression, sleep disturbances, and pain were measured pre and post intervention. Afterwards, the variables were compared in each group and between the groups.

**Results:** Results of study showed that the ginseng improved the quality of life and mood in the subjects. (P<0.0001) and no difference was observed in the placebo group (P=0.887).

**Conclusion: **The use of ginseng may can effective on reducing CRF and the associated symptoms in the patients with cancer, but further studies should be conducted for the evaluation of comprehensive therapeutic efficacy.

## Introduction

 Ginseng is generally known as red Panax ginseng. The plant of ginseng is from Aralicaceae family which grows in Korea, Northeast China, and Siberia^   1 ^. Ginseng root is a common Chinesemedication, believed to have vitality-enhancing properties. It is believed to improve appetite, fortify the immune system, mitigate pain and headache, enhance cognitive functioning and performance status and treat depression. The mechanisms of action of ginseng are fully understood. However, it has been reported that it influences corticotrophin, cortisol, immune response adjustment, antioxidant production, neuroendocrine activities, metabolism of carbohydrates and lipids, and the nitric acid production in cardiovascular system  2^,^^3^ . There are two types of ginseng: Panax quinquefolius and Panax ginseng both contain active pharmacological components called ginsenosides that have anti-inflammatory characteristics ^4^^-^^6^ . Ginsenosides are a class of triterpene saponins. The ginseng root also contains non-saponin components such as polyacetylene, fenols, sesquiterpenes, alkaloids, oligosaccharides and aminoglycosides ^1^^,^^3^^,^^7^ . Cancer-related fatigue (CRF) significantly affects the quality of life (QOL) of cancer patients through limiting the daily activities of the patients ^8^^,^^9^ . Previous studies have demonstrated that the debilitating symptoms of cancer and the reduced physical performance remain even after cancer treatment. The fatigue and lack of vitality are among the most common symptoms in cancer patients. It is estimated that 60-90% of patients with cancer experience CRF ^10^^,^^11^. It affects the functions and performance of the patient’s brain through direct and indirect mechanisms and factors. Moreover, other factors such as anxiety, sleep disturbances, pain and lack of appetite could exacerbate the fatigue and disabling condition in the cancer patients ^11^^,^^12^ . Considering the impact and significance of CRF, developing new strategies to manage and reduce this symptom is necessary. It has been reported that the cancer patients experience fatigue in both short-and long-term daily activities 8^,^^9^^,^^13^^-^^15^ . The fatigue, faint, and disability are observed in 59 % of cancer patients who finished their chemotherapy sessions and 65-100 % of the patients who are under chemotherapy treatment. Researchers have concluded that CRF could persist after 5 to 10 years after the end of chemotherapy^13^^,^^15^. Using food supplements is a self-cure for CRF in the patients with cancer. The efficacy of the Q10 coenzymes, L-Carnitine, Guar and Ginseng in reducing CRf has been studied and the findings showed that Q10 coenzymes and L-carnitine showed no superiority over the placebos ^16^^,^^17^ . Ginseng is known as an adaptogen in Chinese traditional medicine^   18 ^^.^ The mechanism of action of American Ginseng which mitigates fatigue is not yet known, but the studies have suggested that it may act through influencing the activity of hypothalamic axis of the hypophysis gland in reducing the chronic fatigue ^19^^-^^21^ .

Previous studies have demonstrated that ginseng decreases the inflammation^   22 ^ and the chronic stress on the hypothalamic axis of the hypophysis gland ^4^^-^^6^ . No toxicity or side effect has been reported by the patients who took considerable amount of ginseng^   23 ^. The aim of the present study was to evaluate the efficacy and safety of Panax ginseng (PG) in reducing CRF in patients with non-metastatic cancer.

## MATERIALS AND METHODS

 This was a prospective clinical trial conducted on the patients (n=113) suffering from non-metastatic colon cancer (age range: 20-70 years old) referring to the Shafa Hospital, Ahvaz, Iran for chemotherapy treatment. After the enrollment of the patients and before the start of the study, all study procedures, the main objectives, possible benefits and side effects were clearly explained to the patients. Then, all patients read and signed the written informed consent form indicating their willingness to participate in the study. After the chemotherapy sessions, the patients were randomly divided into two groups. The first group received daily dose of 100 mg PG and the second group received placebo medication. The demographic information and clinical parameters of patients, including age, sex, weight, symptoms of fatigue, depression, sleep disturbances, and pain were measured pre and post intervention and the variables were then compared in each group and between the groups. 

The experimental procedures of this study including the intervention, clinical assessment, and data collection were performed at Shafa Hospital, which is affiliated to Ahvaz Jundishapur University of Medical Sciences (AJUMS), Ahvaz, Iran. All of the protocols of this study were approved by the local Ethics Committee of AJUMS, Ahvaz, Iran (Registration code: ajums. rec. 1392. 323) which were in complete accordance with the ethical regulations of human studies set by the Helsinki declaration (2014). The study was registered in the Iranian Registry of Clinical Trial. A customized questionnaire used to collect the variables consisted of two parts: BEK test and the researcher-built test. The short-form inventory for assessing recording the symptoms of fatigue included three questions based on QOL (muscular pain, degree of happiness, and sleep quality). The answers were coded on the scale of 1 to 3 (mild, moderate, and severe). The second part was the BEK Test which was answered by the patients before and after administration of ginseng and placebo capsules.

Mood conditions (mild, medium, and increased), sleeping ability (good, relatively difficult, with difficulty) and BEK test (a: good, B: depression and C: severe depression D: clinical treatment required) were asked by the researcher and check-marked in the questionnaire.

The capsules of the ginseng extract, Panax ginseng (PG) were produced with the health code 302002039014 (Ginsana Co Switzerland) and were available in the market. The procedures of this study were performed with full observation of the protocol set forth by the Medical Ethics Committee and with conscious consent of participants. Those patients who took at least 25 out of 30 capsules were eligible to enter into the study. The age and ethnicity of the metastasis and non-metastasis groups were other criteria; the metastasis group was assigned to receive only a single cycle of chemotherapy. The capsules were taken in the morning after breakfast, and the patients should not have a history of controlled high blood pressure, tachycardia, or previous consumption of ginseng medications. Those who did not meet these criteria were excluded from the study. After the study period, the patients were again assessed with respect to BEK Test. According to the WHO reports, the recommended dose of ginseng is up to 3 g of the root of the ginseng plant which contains 4 to 7 mg of ginsenosides ^24^^,^^25^. Several clinical tests on the dose range of 40-800 mg and 2-6 g of the root of ginseng used by the patients have shown no toxicity ^   26 ^. In this study, a daily dose of 100 mg (one capsule) for 30 days was administered to the patients every morning after breakfast.

## Results

 A total of 114 cancer patients (age range: 20-70 years) participated in this study and were divided into treatment group (n=54) and placebo group (n=60). The demographic information of the patients in the two groups of intervention and placebo are presented in Table 1.

**Table 1:** The demographic information of the patients participated in the study

**Variable**		**Ginseng**	**Placebo**	**P-Value**
		**Mean±SD**	**Mean±SD**	
Age		50.11±10.459	48.03±10.556	0.292
Weight		63.74±8.450	63.38±8.301	0.817
		N (%)	N (%)	P-Value
Gender	Men	28 (51.85%)	31 (51.67%)	0.894
	Women	26 (48.15%)	29 (48.33%)	

In the beginning of the study, a comparison of the variables through Chi-square test showed no significant difference between the groups indicating that the two groups were statistically matched (P-value> 0.05).

The questionnaires were again completed for the same variables after the intervention and the result of comparison showed improvement of the mood. BEK test showed better sleeping abilities in the patients taking ginseng (P-value<0.0001). During the pre-intervention assessment by the BEK questionnaire, the majority of patients in the intervention group showed the moderate level of depression, whereas the vast majority of all age groups rated good or no depression after the intervention. In the placebo group, the majority of the age groups showed the moderate level of depression before and after the intervention. The results showed that ginseng was effective in improving depression and the quality of life in the intervention group (Table 2).

**Table 2:** Results of Beck questionnaire before and after the intervention

**Groups**		**Beck**		**P-Value**
		**Before** **N (%)**	**After** **N (%)**	
Ginseng	Good	13 (24.07%)	44 (81.48%)	<0.0001^*^
	Depression	30 (55.56%)	10 (18.52%)	
	Severe depression	9 (16.67%)	0 (0%)	
Placebo	Good	13 (21.67%)	11 (18.33%)	0.887
	Depression	34 (56.66%)	39 (65%)	
	Severe depression	13 (21.67%)	10 (16.67%)	

## Discussion

 The studies have shown that ginseng has no interference with chemotherapy medications such as tamoxifen, doxorubicin, cyclophosphamide, fluorouracil and methotrexate ^22^^,^^27^ . However, further studies should be conducted in this regard in the future. The findings of this study demonstrated that the ginseng has anti-fatigue properties. However, the significant clinical effects of ginseng will not be discernible in the first week of consumption and the effect will appear at least following one month. Better benefits in patients undergoing cancer treatment might imply that ginseng may act as a preventive factor of CRF during the treatment. This study also demonstrated that high dosage intake of PQ was tolerable and no adverse consequences were reported ^26^^-^^28^. In addition, our findings showed that the symptoms of CRF including pain, appetite and QOL were significantly improved following the administration of a daily 100 mg ginseng for 30 days. In a double-blinded clinical trial, Barton et al. tested American ginseng in three doses of 750 mg, 1000 mg, and 2000 mg and the results demonstrated a significant improvement in the CRF conditions compared with the placebo patients. Meanwhile, no toxicity was reported^29^. A similar study reported that ginseng extract had anti-depression effects on liver in rats. This antidepressive effect might be similar to the effects of fluoxetine through inhibiting the absorption of serotonin. The results of the study on 50 cancer patients showed that taking 3 g of sun ginseng for 12 weeks significantly improved the physical activities and psychological aspects of the patients^30^.

In this study which was aimed at investigating the effects of ginseng consumption in the improvement of the QOL in patients suffering from colon cancer, considerable improvement of the mood and the psychological states were observed, whereas the sleeping ability of the patients was worsened which can be attributed to the improvement in the patients’ mood. Non-randomized design was the main procedure used in the data collection for the QoL assessments. Therefore, the data on the QOL were removed from the study. In addition, both ginseng administration and follow-up periods were relatively short. Therefore, the findings of this study cannot be generalized without caution. Conducting similar studies with big sample size and in random design is suggested. 

## CONCLUSION

 The findings of this study encourage the use of ginseng for reducing CRF and the associated symptoms. However, further studies should be conducted to reach a decisive conclusion on the therapeutic efficacy of ginseng and mechanisms of action.
